# Conversion of dendritic cells into tolerogenic or inflammatory cells depends on the activation threshold and kinetics of the mTOR signaling pathway

**DOI:** 10.1186/s12964-024-01655-1

**Published:** 2024-05-21

**Authors:** Viktor Wixler, Yvonne Boergeling, Rafael Leite Dantas, Georg Varga, Stephan Ludwig

**Affiliations:** 1 Institute of Molecular Virology, Centre for Molecular Biology of Inflammation (ZMBE), Westfaelische Wilhelms- University, Von-Esmarch-Str. 56, 48149 Muenster, Germany; 2 Pediatric Rheumatology and Immunology, University Children’s Hospital Muenster, 48149 Muenster, Germany; 3 Present Address: Department of Mental Health, Westfaelische Wilhelms-University, 48149 Muenster, Germany

**Keywords:** Dendritic cells, mTOR signaling cascade, Tolerogenesis, Immunogenesis, Peripheral immune tolerance, Small spleen peptides

## Abstract

**Background:**

Restoring impaired peripheral immune tolerance is the primary challenge in treating autoimmune diseases. Our previous research demonstrated the effectiveness of small spleen peptides (SSPs), a fraction of low molecular weight proteins, in inhibiting the progression of psoriatic arthritis, even in the presence of high levels of the proinflammatory cytokine TNFα in the bloodstream. When specifically targeting dendritic cells (DCs), SSPs transform them into tolerogenic cells, which efficiently induce the development of regulatory Foxp3^+^ Treg cells. In this study, we provide further insights into the mechanism of action of SSPs.

**Results:**

We found that SSPs stimulate the activation of the mTOR signaling pathway in dendritic cells, albeit in a different manner than the classical immunogenic stimulus LPS. While LPS-induced activation is rapid, strong, and sustained, the activity induced by SSPs is delayed, less intense, yet still significant. These distinct patterns of activation, as measured by phosphorylation of key components of the pathway are also observed in response to other immunogenic and tolerogenic stimuli such as GM-CSF + IL-4 or IL-10 and TGFβ. The disparity in mTOR activation between immunogenic and tolerogenic stimuli is quantitative rather than qualitative. In both cases, mTOR activation primarily occurs through the PI3K/Akt signaling axis and involves ERK and GSK3β kinases, with minimal involvement of AMPK or NF-kB pathways. Furthermore, in the case of SSPs, mTOR activation seems to involve adenosine receptors. Additionally, we observed that DCs treated with SSPs exhibit an energy metabolism with high plasticity, which is typical of tolerogenic cells rather than immunogenic cells.

**Conclusion:**

Hence, the decision whether dendritic cells enter an inflammatory or tolerogenic state seems to rely on varying activation thresholds and kinetics of the mTOR signaling pathway.

**Supplementary Information:**

The online version contains supplementary material available at 10.1186/s12964-024-01655-1.

## Background

Dendritic cells (DCs) are professional antigen-presenting cells that play a central role in initiating both the adaptive immune responses, inflammation and immune tolerance. While immunogenic DCs trigger immune responses against foreign antigens by presenting them to T cells and stimulating their activation and differentiation into effector T cells, tolerogenic DCs foster immune tolerance by promoting the generation of regulatory T cells (Tregs) and prevent harmful autoimmune responses. Hence, the balanced differentiation of immunogenic and tolerogenic DCs ensures a well-functioning immune system, protecting the body against external invaders and autoantigens.

Similar to other cells, the differentiation of immature DCs into specialized immunogenic or tolerogenic cells is governed by specific stimuli that activate intracellular signaling pathways. In this context, the mTOR (mammalian target of rapamycin) signaling pathway plays a central role in the development and function of both immunogenic and tolerogenic DCs [1–3]. It is widely assumed that mTOR activation in DCs is associated with immunogenic responses, while mTOR inhibition can promote the generation of tolerogenic DCs [3, 4].

The mTOR pathway consists of two major protein complexes, mTORC1 and mTORC2, each with distinct regulatory proteins but both containing the serine/threonine kinase mTOR as a central actor [3–6]. mTORC1 is sensitive to the antifungal antibiotic rapamycin (from which the name mTOR is derived), while mTORC2 is insensitive [5]. Interestingly, rapamycin has been shown to suppress Th1 immune responses in animal models and stimulates the proliferation of immunosuppressive regulatory Treg cells [7–9].

The mTOR pathway is a multifunctional signaling cascade that not only modulates the immune response but also participates in various other cellular processes. Accordingly, its activity is regulated by various upstream signals, including growth factors, nutrients, energy levels and cellular stress [3, 5, 10]. However, the functional consequences of mTORC1 and mTORC2 activation are somewhat different [1]. mTORC1 exerts its function by controlling translation, transcription, ribosome biogenesis, nutrient transport and autophagy. The mTORC2 complex, on the other hand, controls cell proliferation and survival by organizing the actin cytoskeleton and activating Akt. A pivotal event in mTOR signaling pathway activation is the phosphorylation and activation of the downstream ribosomal protein S6 kinase beta-1 (p70S6K), which plays a critical role in protein synthesis, ribosome biogenesis, and cell growth and differentiation [1, 5, 11].

In a recent study, we demonstrated that small spleen peptides (SSPs), a fraction of low molecular weight proteins isolated from the spleen, can restore impaired peripheral immune tolerance in vivo and identified DCs as the primary targets of SSPs. These peptides induced a tolerogenic state in immature DCs, enabling them to efficiently induce the development of Foxp3^+^ immunosuppressive Treg cells [12]. Thus, to explore the molecular mechanisms underlying the tolerogenic transformation of DCs by SSPs, we investigated here the cellular kinase activities in DCs after peptide stimulation and found the phosphorylation of the mTOR downstream effector p70S6K to be one of the top hits. Hence, we examined the role of mTOR signaling in the SSP-mediated tolerogenic induction of immature DCs by comparison with classical immunogenic (LPS, GM-CSF + Il-4) and tolerogenic (IL-10 and TGFβ) stimuli.

## Materials and methods

### Mice, cells and reagents

All mice were bred and housed in a specific pathogen-free facility at the University of Muenster, in full compliance with the German regulations set forth by the Society for Laboratory Animal Science (GV-SOLAS) and the European Health Law of the Federation of Laboratory Animal Science Associations (FELASA). The experimental protocols conducted in this study were approved by the Landesamt für Natur, Umwelt und Verbraucherschutz Nordrhein-Westfalen (LANUV-NRW), Germany.

Bone marrow cells were extracted from the femur and tibia of C57Bl/6 (H-2^b^) mice using PBS/1% FCS (Biotrend Chemikalien GmbH, Cologne, Germany). The erythrocytes were then lysed using hypo-osmolar ACK lysing buffer from Thermo Fisher Scientific (Schwerte, Germany) for 3 min at room temperature. After filtering the remaining cells through a 40 μm cell strainer and washing them with PBS, the cells were seeded in 6-well plates (not tissue culture treated) at a concentration of 0.6 × 10^6^ cells/ml in 5 ml RPMI 1640 medium (Sigma Aldrich, Taufenkichen, Germany) supplemented with 10% FCS (fetal calf serum), 1% L-glutamine, 1% non-essential amino acids, 1% sodium pyruvate, and 50 µg/ml gentamycin. To mature the bone marrow-derived cells into DCs, GM-CSF + IL-4 (10 ng/ml each) was added. On day 3 of maturation, half of the medium in the dishes was replaced with fresh medium, and the cells were further cultivated until day 6.

All cytokines were purchased from BioLegend® (Fell, Germany) and LPS from E.coli 055:B5 from Sigma (Taufenkichen, Germany). SSP samples were isolated either from porcine or mouse spleen as described previously [12]. The SSP isolation procedure is patented under the number WO 2022/173,328 A1 from 18 February 2022. All peptide samples used in this work were classified as LPS-free according to the LAL test (Sigma, Taufenkirchen, Germany). In general, samples containing less than 2 pg/µg were considered LPS-free. The antibodies used for Western blots were from Cell Signaling Technology (Leiden, The Netherlands) and the kinase inhibitors, from Tocris Bioscience (Wiesbaden, Germany). The adenosine receptor inhibitors, including CGS15943 (pan inhibitor), DPCPX (A1R inhibitor), ZM241385 (A2AR inhibitor), and MRS1706 (A2BR inhibitor), were obtained from Tocris. Additionally, the adenosine A3R inhibitor, MRS1523, was sourced from Biozol (Eching, Germany). The optimal concentrations to use were determined through preliminary experiments.

### Stimulation of dendritic cells for Western blot analysis

On day 6 of maturation with GM-CSF + IL-4, DCs were washed twice with PBS/1% FCS and adjusted to a concentration of 1 × 10^6^/ml in RPMI/1% FCS medium. Four milliliters of the cell suspension were transferred into 5 ml Eppendorf tubes and subjected to a starvation period of 6 hours with rotation at 5 rpm/min at 37 °C. Following the starvation period, DCs were stimulated with SSPs (5 µg/ml), IL-10 (50 ng/ml), TGFβ (50 ng/ml), GM-CSF + IL-4 (10 ng/ml each), or LPS (50 ng/ml) for various durations while being rotated at 37 °C. The optimal concentrations for the stimuli listed here were determined in preliminary tests based on the ability to convert DCs into an immunogenic or tolerogenic state [12]. When kinase inhibitors were utilized, they were added to the starved DCs 30 min prior to stimulation with SSPs or LPS and remained present throughout the entire stimulation period.

Following stimulation, the cells were centrifuged at 350 g for 3.5 min, and the resulting pellets were lysed in 200 µl of M-PER lysis buffer supplemented with 1x Halt Phosphatase and Protease inhibitor cocktails from ThermoFisher for 10 min on ice. After clarifying the lysates through centrifugation at 15,000 g at 4 °C for 10 min, each sample containing 20 µg was separated using 7.5–10% SDS-PAGEs. Subsequently, the proteins were transferred to a nitrocellulose membrane and detected using appropriate antibodies, along with horseradish peroxidase-conjugated secondary antibodies and the ECL detection system. Each nitrocellulose membrane was utilized to analyze 5 to 7 distinct proteins. We performed sequential Western blotting without stripping the antibodies from previous blots. However, we ensured that the proteins being analyzed were not in close proximity to each other in terms of their molecular weight and subsequent position on the membrane. The order of protein detection on the respective membranes did not always follow the same sequence as depicted in the figures of this manuscript. Nevertheless, the underlying principle that guided our approach is illustrated in Additional file 1 (Figure S3), which includes complete, uncut blot images.

### Kinase activity profiling

Following stimulation with SSPs for the indicated times, DCs were washed with PBS through centrifugation, and the resulting pellets were lysed using ice-cold M-PER Mammalian Extraction Buffer (Thermo Fisher Scientific) supplemented with Halt Phosphatase Inhibitor Cocktail and EDTA-free Halt Protease Inhibitor Cocktail (100×, Thermo Fisher Scientific). After centrifugation at 15,000 g for 10 min at 4 °C, the supernatants were frozen at -80 °C until further processing. Protein concentrations were determined using the Pierce BCA protein assay (Thermo Fisher Scientific) according to the manufacturer’s instructions. For the protein tyrosine kinase (PTK) array protocol (v04), 5 µg of protein extract was utilized, while 0.5 µg was used for the serine/threonine kinase (STK) array protocol (v11). The measurements were conducted on a PamStation®12 from PamGene as previously described [13]. The PTK assay involved a one-step reaction, where cell extracts, ATP, and FITC-labeled pY20 antibodies were incubated on the chip, and the phosphorylation of individual Tyr-peptides was monitored in real time using fluorescence detection. The STK assay, on the other hand, was a two-step reaction. In the first step, cell lysates, ATP, and the primary antibody mixture were incubated on the chip for 110 min, followed by the addition of secondary FITC-labeled antibodies. The development of the fluorescence signal was detected using Alexa488 fluorescence. Signal intensities and their correlation to kinase activity were analyzed using BioNavigator6 v06.03.63.0 (PamGene). Kinome trees were generated using the Coral web application (http://phanstiel-lab.med.unc.edu/CORAL/), which was developed by Katie Metz, Erika Deoudes, Matt Berginski, Arman Aksoy, Ivan Jimenez-Ruiz, and Doug Phanstiel in the Phanstiel Lab at UNC. The Coral application employs phylogenetic information derived from Manning et al. [14].

### Nuclear/cytosolic fractionation

Preparation of cytosolic and nuclear fractions was performed as described previously [15]. DCs were washed twice with ice-cold PBS and cell pellets obtained by centrifugation (5 min 650 g at 4 °C) were lysed in 500 µl Roeder A buffer (10 mM HEPES pH 7.9, 1.5 mM MgCl_2_, 10 mM KCl supplemented with 0.5 mM DTT) and incubated on ice for 10 min. Subsequently, NP-40 was added to a final concentration of 0.3% and cell lysates were incubated for additional 10 min on ice. Next, nuclei were sedimented by centrifugation (10 min at 2650 g and 4 °C) and the supernatant was collected (referred as cytoplasmic fraction). The pellet was resuspended in 50 µl of Roeder C buffer (25% (v/v) glycerol, 0.3 M NaCl, 1.5 mM MgCl2, 20 mM HEPES pH 7.9 supplemented with 0.5 mM DTT). After over-night incubation in an overhead rotator at 4 °C, nuclear fractions were clarified by centrifugation (30 min, 20,000 g at 4 °C) and used for SDS-PAGE and Western blotting.

### Real-time metabolic assay

The oxygen consumption rate (OCR, pmol/min) and extracellular acidification rate (ECAR, mpH/min) of DCs were measured using the Agilent Seahorse XFe96 Extracellular Analyzer (Seahorse Bioscience). For that, bone marrow cells were matured to DCs with GM-CSF + IL-4 for 6 days and after washing off the cytokines, they were further stimulated with IL-10 (50 ng/ml), TGFβ (50 ng/ml), GM-CSF + IL-4 (10 ng/ml each), LPS (10 ng/ml), or SSPs (5 µg/ml) isolated from either porcine (SSP-S10) or murine (SSP-M14) spleen for an additional 48 h. The cells were stimulated in 24-well plates with 1 × 10^6^ cells in 2 ml RPMI/10% FCS per well. After harvesting and washing the cells twice, 15 × 10^4^ cells resuspended in 100 µl of XF Base Medium were applied to poly-D-lysine precoated XF96 flat-bottom cell culture microplates (Seahorse Bioscience). The plates were then briefly centrifuged at 1200 rpm and incubated at 37 °C for 1 h. The XF Base Medium was supplemented with glucose (10 mM), pyruvate (1 mM), and glutamine (2 mM), with pH being adjusted to 7.4.

The OCR and ECAR values were measured simultaneously and the measurements were performed in parallel with all DC samples in an XF96 well microplate. For OCR measurements, four consecutive stages were analyzed: basal respiration (no inhibitors), inhibition of mitochondrial complex V or minimum oxygen consumption (2 µM oligomycin), induction of maximal respiration rate (1.5 µM carbonyl cyanide-4 (trifluoromethoxy) phenylhydrazone (FCCP)), and inhibition of the electron transport chain or non-mitochondrial oxygen consumption (0.1 µM rotenone + 1.0 µM antimycin). For ECAR measurements, three stages were analyzed: basal stage (no drugs), maximal glycolysis induction or glycolytic capacity (2 µM oligomycin), and inhibition of glycolysis or non-glycolytic acidification (50 mM 2-deoxyglucose (2DG)).

### Statistical analysis

Data is expressed as mean ± SEM. Statistical analysis was performed using GraphPad Prism software (version 6). One-way ANOVA followed by Tukey´s comparison analysis was used when more than two groups were compared and 2-way ANOVA, followed by Tukey´s multiple comparisons test when more than one parameter changed in the groups. Results were considered statistically significant at *P* < 0.05 and displayed as * *P* < 0.05, ** *P* < 0.01, *** *P* < 0.001.

## Results and discussion

### Stimulation of DCs with SSPs activates the mTOR signaling cascade

**Fig. 1:**
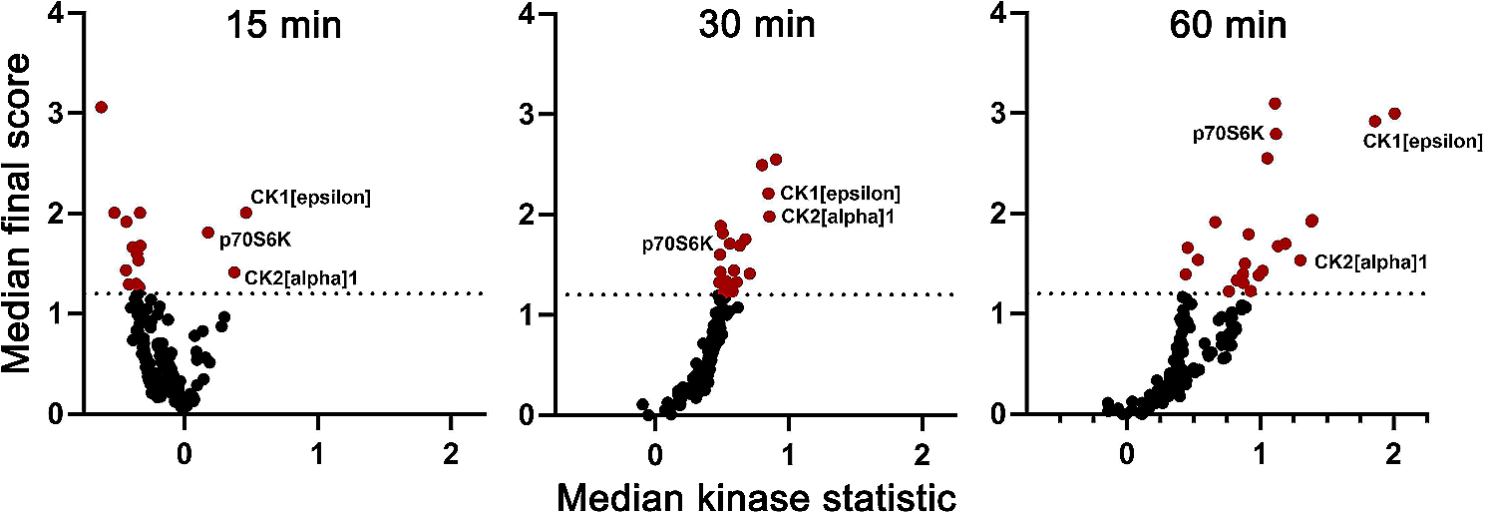
Kinase activation profile in DCs after stimulation with SSPs Kinase activity was assessed by chip-based kinase activity profiling (PamGene technology). The variances in kinase activity of DCs that have been stimulated with SSPs for 15, 30, or 60 min, in comparison to unstimulated control cells (0 min), are illustrated as the median kinase statistics (negative values = lower activity, positive values = higher activity) from three independent experiments. Kinases with high sensitivity (median final score > 1.2) are indicated in red

Since our primary focus is to uncover the molecular mechanisms behind the tolerogenic enhancement of DCs by natural SSPs, we conducted kinase activity profiling assays on PamChip arrays using the PamStation 12 instrument to identify the kinases in DC lysates that are involved in SSP-mediated stimulation. The PamChips contain 144 synthetic peptides for serine/threonine kinases and 196 substrates for tyrosine kinases. Bone marrow cells were cultured with GM-CSF + IL-4 for 6 days to generate semi-mature DCs and following removal of the cytokines, the cells were starved for 6 h in RPMI medium with 1% FCS. Afterward, the cells were subjected to SSPs for 15, 30, and 60 min, and cell lysates were examined in comparison to unstimulated cells or starved cells. Three separate and repeated experiments were conducted for each time point. Notably, the screening yielded a significant activation state-specific phosphorylation at all three time points analyzed for three kinases: ribosomal kinase p70S6K and casein kinases 1 and 2 (Fig. 1, positive median kinase statistic). A detailed phylogenetic tree of the protein kinases identified in the DC lysates as well as the variance between the different replicates of the three independent experiments are shown in Fig. S1 (Additional file 1).

The ribosomal kinase p70S6K is a serine/threonine kinase, also known as ribosomal protein S6 kinase beta-1 (S6K1), and a key downstream substrate for mTOR kinase. It primarily targets the 40 S ribosomal protein S6, whose phosphorylation promotes protein synthesis on the ribosome, and consequently, cell growth and proliferation [16]. The mTOR signaling pathway is known as central integrator of many signaling pathways [6, 10, 17] and given its central role in immunogenesis and tolerance development, we decided to investigate the phosphorylation and activation of different components of the mTOR signaling cascade in more detail. Furthermore, to determine whether SSP-induced stimulation of the mTOR cascade differ from immunogenic but explicitly from other tolerogenic stimuli, we compared the SSP-mediated mTOR signaling with that induced by LPS, GM-CSF + IL-4, TGFβ and IL-10, the classical immunogenic and tolerogenic stimuli, respectively.

**Fig. 2:**
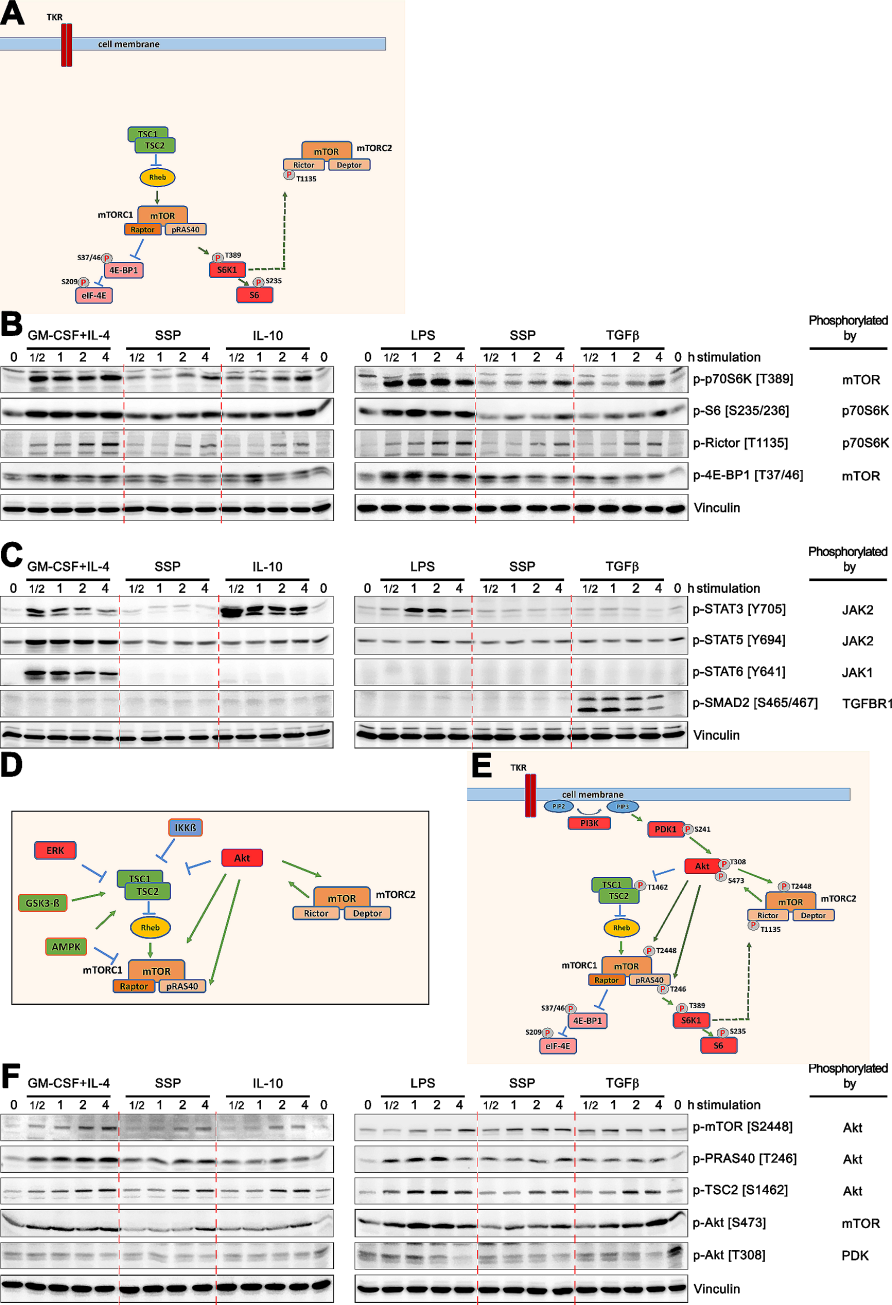
Analysis of the mTOR signaling cascade: exploring the p70S6K and Akt axes (**A**) A schematic representation of a part of the mTOR cascade, containing the signaling pathway components analyzed in (B). This is a simplified representation of the cascade, which mainly shows the components of the signaling pathway relevant to this work. Green lines with arrows indicate activating phosphorylations, while blue truncated lines represent inhibitory phosphorylations. Solid lines represent direct phosphorylation, while dashed lines indicate feedback phosphorylation. (**B**) Phosphorylation of the components of the mTOR signaling cascade at specific sites (indicated on the right) in DCs after their treatment with immunogenic (GM-CSF + IL4, LPS) and tolerogenic (IL-10, TGFβ) stimuli compared to stimulation with SSPs (indicated on the top). To facilitate a better comparison, samples of SSP-stimulated cells were applied on a gel between samples stimulated with immunogenic and tolerogenic agents. The kinase responsible for phosphorylation is always indicated on the far right. The vinculin blot served as a loading control. Representative blots from three to four independent experiments are shown throughout. (**C**) Control phosho-blots, which show the specificity and functional activity of the active substances investigated. (**D**) The schedule represents the main intracellular upstream kinases known to regulate the mTOR cascade. (**E**) An extended scheme of the mTOR signaling cascade with components whose phosphorylation was investigated in (F). (**F**) Phospho-blots that were conducted to investigate the phosphorylation of mTOR signaling pathway components, as indicated on the right, after stimulations with different agents, as indicated on the top

To achieve this, bone marrow cells were cultured with GM-CSF + IL-4 for 6 days to produce semi-mature DCs. Following the removal of the cytokines, the cells were starved for 6 h in RPMI medium with 1% FCS and subsequently exposed to SSPs and either tolerogenic (IL-10, TGFβ) or immunogenic (GM-CSF + IL-4, LPS) stimuli for varying durations. Cell lysates were then examined for the phosphorylation of different components of the mTOR signaling pathway (Fig. 2A) using Western blotting. In order to facilitate a more effective comparison between the effects of SSP and those of immunogenic and tolerogenic stimuli, lysates from DCs stimulated with SSP were consistently loaded onto the gel alongside samples from cells subjected to immunogenic and tolerogenic stimulation (Fig. 2B). This approach also enabled repeated SSP stimulation to be conducted simultaneously.

As anticipated, both GM-CSF + IL-4 and LPS, as immunogenic stimuli, elicited robust phosphorylation of p70S6K at T389 (threonine 389), the site of mTOR kinase phosphorylation. This phosphorylation was consistently evident and persisted throughout the four-hour duration of the study (Fig. 2B). Notably, both tolerogenic stimuli, IL-10 and TGFβ, also promoted phosphorylation of p70S6K kinase at T389, but the timing and intensity of phosphorylation differed from those induced by the immunogenic stimuli. The phosphorylation occurred later and was notably weaker than that following LPS or GM-CSF + IL-4 stimulation, yet it remained reproducible and distinct. It is worth mentioning that the phosphorylation pattern of p70S6K kinase after SSP stimulation closely resembled that of IL-10 and TGFβ stimulation, indicating its tolerogenic rather than immunogenic nature.

To validate the functionality of p70S6K, we further examined the phosphorylation of the ribosomal 40 S protein S6 and the mTORC2 subunit Rictor, both of which are substrates of p70S6K (Fig. 2A). Indeed, both physiological substrates of p70S6K were phosphorylated at sites specific to the kinase, following a pattern similar to that observed for p70S6K activity. Immunogenic stimuli, GM-CSF + IL-4 and LPS, induced p70S6K-dependent S6 and Rictor phosphorylation earlier and more intensely than the tolerogenic IL-10 and TGFβ stimuli, including SSPs, which exhibited delayed and less intensive phosphorylation (Fig. 2B).

Rictor is a scaffold of the mTORC2 protein complex, maintaining its structure and exerting negative regulation on its activity (Fig. 2A). Phosphorylation of Rictor at T1135 abolishes its inhibitory function on mTORC2, resulting in an increased kinase activity of mTORC2 and thus in an elevated mTORC2-dependent phosphorylation of Akt and its downstream substrates [18]. Rictor thus functions as part of a negative feedback mechanism that controls Akt activity (Fig. 2D and E). Phosphorylation of the ribosomal S6 protein at S235/236 in turn has several functional consequences, but is primarily associated with an increased protein synthesis [5, 19].

Another crucial component of the mTOR cascade, situated downstream of the mTORC1 complex and contributing significantly to the regulation of protein synthesis, is the 4E-PB1 – eIF-4E branch (Fig. 2A). Phosphorylation of the 4E-PB1 protein by mTOR prevents its interaction with its binding protein, eukaryotic initiation factor 4E (eIF-4E), ultimately facilitating cap-dependent translation [20]. Consequently, the phosphorylation of p70S6K and 4E-PB1 by mTOR results in increased mRNA biogenesis, cap-dependent translation and elongation, as well as the translation of ribosomal proteins [5]. As expected, the phosphorylation of 4E-PB1 at T37/46, the sites previously shown to be targeted by mTOR kinase, was also detectable following all the stimuli tested, with greater intensity observed after immunogenic stimuli (Fig. 2B).

In conclusion, these findings demonstrate that the downstream effectors of the mTOR kinase are phosphorylated and activated by all the immunogenic and tolerogenic stimuli examined, including SSPs, indicating that mTOR is a central regulatory kinase to all stimuli. Indeed, p70S6K and 4E-PB1 are direct substrates of mTOR. However, the timing and intensity of their phosphorylation by these two distinct types of stimuli varied. Immunogenic stimuli induced rapid, intense, and prolonged phosphorylation of members of the mTOR pathway, whereas tolerogenic stimuli elicited a delayed and less intense response. This differential pattern of phosphorylation/activation in response to the two types of stimuli was consistent across the different components of the mTOR cascade studied with both positive and negative regulatory functions, suggesting a universal yet specific activation of the pathway by the immunogenic and tolerogenic stimuli. Notably, DCs stimulated with SSPs consistently exhibited behavior akin to that of tolerogenic DCs in terms of the induction of phosphorylation of components within the examined mTOR cascade.

### Verification of stimuli specificity

As all tested stimuli activate the mTOR pathway, our next step was to verify the functionality and specificity of the cytokines and other stimulants used. To achieve this, we examined DC lysates for the phosphorylation of specific proteins known to be selectively phosphorylated and activated by each respective agent (Fig. 2C). As anticipated, GM-CSF + IL-4 stimulation led to robust phosphorylation of STAT5 (a GM-CSF target) and STAT6 (an IL-4 target), with only minimal phosphorylation of STAT3, which is primarily associated with IL-10 induction. Additionally, phosphorylation of SMAD proteins was observed exclusively following TGFβ application, in line with expectations, while LPS induced STAT3 phosphorylation to a lesser extent than IL-10. Notably, SSPs did not elicit phosphorylation of any of the examined proteins, underscoring its distinctiveness as a tolerogenic inducer and the absence of IL-10 or TGFβ contaminants in SSP preparations.

Collectively, these findings underscore that the observed phosphorylation patterns in DCs following immunogenic and tolerogenic stimuli were not primarily driven by STAT- or SMAD-mediated cascades, despite being induced by certain immunogenic and tolerogenic agents. Furthermore, the use of SSPs, as spleen peptides and natural regulators of tolerogenesis [12], is likely to result in fewer inflammatory side effects when administered in vivo, as they do not elicit the phosphorylation of STAT or SMAD proteins typically associated with inflammation. Nonetheless, they are capable of specifically activating the mTOR signaling pathway to facilitate tolerance formation as effectively as IL-10 or TGFβ.

### Akt kinase is activated via the PI3K/PDK signaling axis by both immunogenic and tolerogenic stimuli

The observation that both immunogenic and tolerogenic stimuli activate the mTOR signaling cascade, but in distinct ways raises important questions. What are the upstream kinases of mTOR that lead to differential activation of its substrates and ultimately result in distinct functional cell specialization? Do these stimuli involve the same kinases, and what determines the diverse modes of mTOR activation?

Among the known main intracellular regulators of mTOR, the PI3K/Akt kinase axis is undoubtedly the most important, followed by ERK, GSK3β, AMPK and IκB kinases (IKK) (Fig. 2D). Most kinases control mTOR kinase activity rather indirectly, either via phosphorylation of the tuberous sclerosis complex (TSC), which is a GTPase-activating protein (GAP) for the small GTPase Rheb and a negative regulator of mTOR, or via the phosphorylation of components within the mTOR complexes. However, Akt kinase can do both: it is able to stimulate mTOR kinase directly by phosphorylating it as well as indirectly through the phosphorylation of TSC or mTOR protein complexes. Phosphorylation of the TSC protein complex can lead to either its inhibition or activation, thereby resulting in the stimulation or suppression of mTOR kinase activity. It is not our aim here to provide a comprehensive overview of the topic; we focus only on selected relevant aspects, but a plethora of excellent reviews on this subject have been recently published [5, 6, 9, 10, 21, 22].

Via the production of phosphatidylinositol (3,4,5)-trisphosphate (PIP3), PI3K recruits PDK1 to the cell membrane, which leads to the phosphorylation of a conserved serine (S241) in the activation loop of PDK1 resulting in its activation [23] and finally in the phosphorylation and activation of Akt (T308). Subsequently, Akt phosphorylates mTOR at S2448, which in turn leads to the phosphorylation of Akt by mTOR at S473 and its activation (Fig. 2E). It is worth noting that mTOR is one of the major kinases that phosphorylates Akt at S473 and that this phosphorylation process is crucial for the full activation of Akt [24]. Indeed, analysis of mTOR phosphorylation revealed that S2448 is phosphorylated after both immunogenic and tolerogenic stimulation of DCs (Fig. 2F), with a kinetic similar to that of p70S6K (Fig. 2B), suggesting the involvement of Akt kinase in the activation of the mTOR pathway.

To validate Akt activation, we investigated the phosphorylation of additional Akt substrates within the mTOR pathway, namely TSC2 and pRAS40 (Fig. 2E and F). Both phosphorylation patterns closely resembled those observed for p70S6K and showed a clear difference between immunogenic and tolerogenic stimulation. While the first phosphorylation event is supposed to activate mTOR by inhibition of the upstream GTPase-activating protein complex TSC1/2, phosphorylation of pRAS40 at T246 leads to its dissociation from the mTORC1 complex and the cessation of its inhibitory activity on mTOR kinase [14, 25]. Finally, we examined the phosphorylation sites of Akt at different sites (Fig. 2F). While phosphorylation at S473 followed a similar kinetic pattern to the other analyzed proteins and was notably prominent after DC stimulation with GM-CSF + IL-4 or LPS, we did not observe significant phosphorylation of Akt at T308.

The failure to detect phospho-T308 raises the question of whether Akt activation occurs through pathways other than PI3K/PDK, or if the used antibody is malfunctioning or the phosphorylation levels are below the detection threshold of the antibody. To address these questions, we treated A549 cells with EGF or insulin, known stimulators of Akt phosphorylation at sites S473 and T308, and conducted a time-dependent analysis of cell samples using antibodies against both phosphorylation sites in parallel. As shown in Fig. S2A (Additional file 1), Akt gets phosphorylated at both sites within as early as 5 min following EGF stimulation. However, the anti-S473 antibody exhibited a significantly stronger and more sustained signal than the anti-T308 antibody. Furthermore, the phosphorylation signal from T308 was not only weaker, but also appeared to wane more rapidly than that from S473.

To finally clarify, whether Akt activation occurs indeed through the PI3K/PDK axis, we stimulated DCs with SSPs or LPS for 4 h in the presence of the highly specific PI3K inhibitor pictilisib and subsequently examined the phosphorylation of p70S6K and Akt kinases. To ensure the absence of any kinase activity in unstimulated cells, the inhibitor was introduced 30 min before the start of the SSP or LPS stimulation. Pictilisib, with an IC50 of 3 nM in cell-free assays, inhibited SSP- and LPS-induced phosphorylation of Akt at S473 by more than 70% already at a concentration of 40 nM, and the inhibition increased with rising concentrations of pictilisib (Additional file 1, Fig. S2B, C). The phosphorylation of p70S6K and TSC2, downstream effectors of Akt, was also significantly diminished in the presence of pictilisib, strongly indicating that the Akt phosphorylation observed here, and consequently mTOR and p70S6K, is mediated through the PI3K/PDK axis. Interestingly, both LPS- and SSP-mediated activation of mTOR components was effectively blocked by pictilisib, although the effects on LPS stimulation appeared to be less pronounced (Additional file 1, Fig. S2B, C).

### ERK1/2 kinases are activated in DCs after both immunogenic and tolerogenic stimulation

**Fig. 3:**
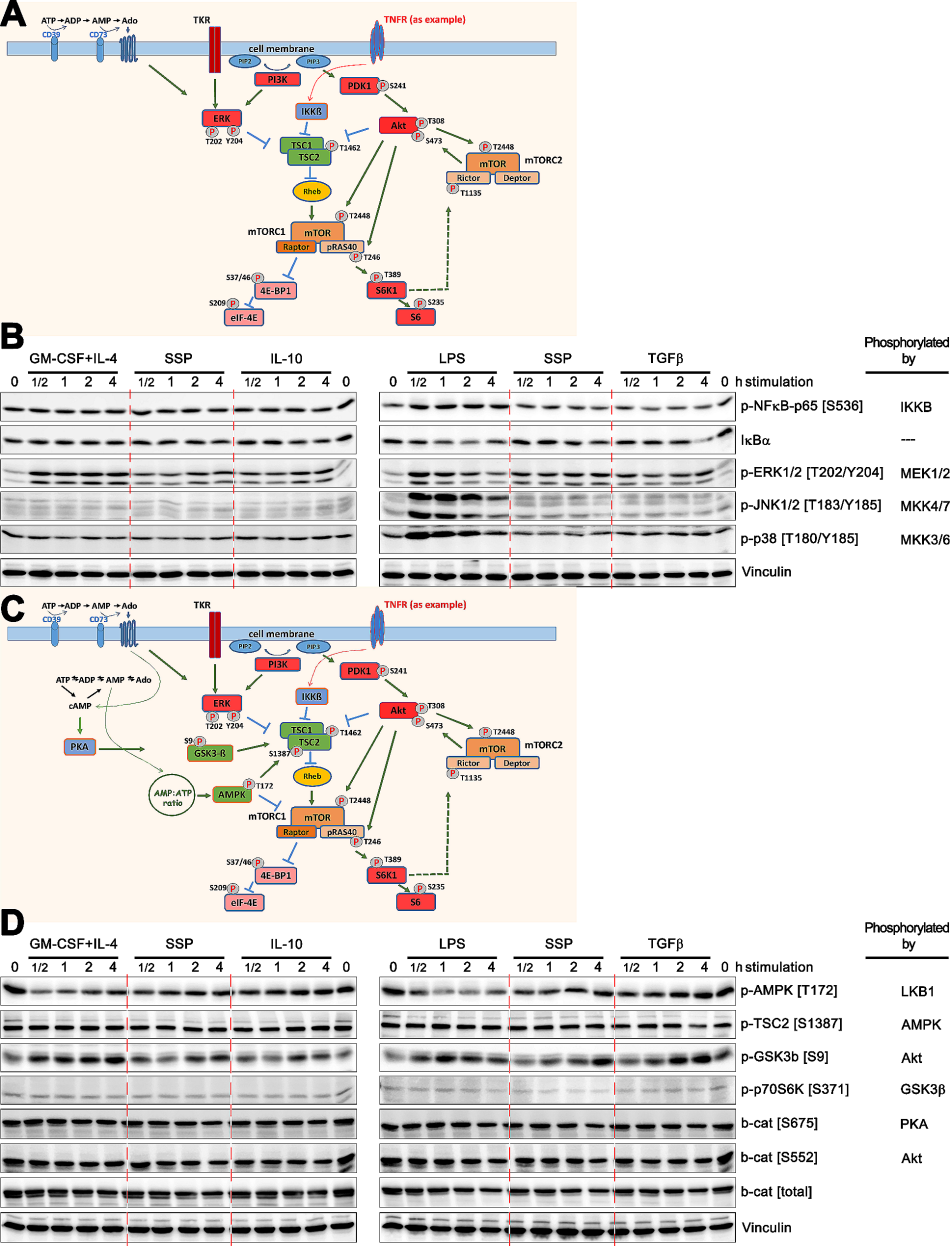
Analysis of the mTOR signaling cascade: exploring the NF-κB, MAPK, AMPK and GSK3β axes (**A**) An extended scheme of the mTOR signaling cascade, including components whose phosphorylation was investigated in (B). (**B**) Phospho-blot analysis to examine the phosphorylation of specific mTOR cascade proteins, as indicated on the right, after different stimulations, as indicated on the top. (**C**) A further extension of the mTOR signaling cascade that includes phosphorylation sites investigated in (D), but it is a simplified representation of the cascade, which mainly shows the components of the signaling pathway relevant to this work. (**D**) Phospho-blots showing the phosphorylation of specific mTOR cascade proteins, as indicated on the right, after different stimulations, as indicated on the top. Representative blots from three to four independent experiments are shown throughout

In a next step, we analyzed whether the induction of the mTOR cascade via NF-κB signaling (Fig. 3A) is significant for the differentiation of DCs into either an immunogenic or tolerogenic lineage. The plausibility of this assumption is supported by the ability of NF-kB signaling to impact mTOR activation, as well as the well-established role of LPS as a potent inducer of IKKs and subsequent inflammatory responses [26, 27].

Phosphorylation analysis of the p65 subunit at S536, a crucial component of the NF-κB complex [28], showed that LPS induced its activation. However, GM-CSF + IL-4 or any tolerogenic stimulus were not capable of its induction (Fig. 3B). Furthermore, the degradation of IκBα and its subsequent resynthesis, which is part of the inhibitory feedback loop of NF-κB activation, was also affected only in response to LPS stimulation. Thus, it appears that NF-κB activation plays a limited role as a decisive trigger for the immunogenic activation of the mTOR cascade, and is not the sole reason for mTOR activation by SSPs.

ERK1/2 kinases, acting as downstream mediators of various extracellular signals and regulators of cell proliferation, differentiation, survival, and immune responses, also have a critical role in modulating the mTOR signaling cascade (Fig. 3A). ERK1/2 kinases can engage with the mTOR pathway through various mechanisms [4, 5, 22]. They facilitate mTORC1 activation by phosphorylating TSC2, leading to the inhibition of TSC1/2 complex and subsequent mTORC1 activation. In addition, ERK1/2 can indirectly affect the mTOR pathway through their downstream effectors, RSK and MK2, resulting in mTOR signaling activation as well [29]. While MK2 is a primary substrate of JNK and p38 kinases, it can also undergo phosphorylation by ERK [30]. Moreover, ERK1/2 is interconnected with the PI3K/Akt/mTOR signaling pathway through mechanisms such as cross-inhibition, cross-activation, and convergence on common substrates, an illustrative example of which is the inhibition of ERK by pictilisib, a specific inhibitor of PI3K (Additional file 1, Fig, S2B and C). Furthermore, ERK1/2 can be activated by other stimuli, such as adenosine receptors, which are known triggers of tolerogenesis [31].

It was therefore important to investigate the activation of ERKs. Conversely to NF-κB, mitogenic ERK1/2 kinases displayed robust and sustained phosphorylation following both LPS and GM-CSF + IL-4 stimulation of DCs, as well as after IL-10 and TGFβ and SSP application, albeit to a lesser extent than with immunogenic stimuli, but still clearly evident (Fig. 3B). Phosphorylation of the other MAPKs, JNK1/2 and p38, which are known for their significant role in inflammation, exhibited strong phosphorylation only following LPS stimulation particularly at the onset of the stimulation and diminished over time. JNK kinases did not exhibit phosphorylation following any stimulus other than LPS, and the p38 kinase displayed minimal to no phosphorylation following GM-CSF + IL-4 and the other tolerogenic stimuli. Consequently, among the analyzed MAPKs, only ERK isoforms, but not JNK or p38, mirrored the phosphorylation and activation pattern of the p70S6K kinase in response to the immunogenic and tolerogenic stimuli examined in this study.

### AMP-activated protein kinase is not activated in DCs after immunogenic or tolerogenic stimulation

Next, we wanted to ascertain whether AMP-activated protein kinase (AMPK) contributes to the activation of the mTOR signaling cascade in DCs in response to the studied stimuli. AMPK is a kinase that inhibits rather than stimulates the mTOR signaling cascade (Fig. 3C), which raises the question of whether it is inhibited in different ways following the stimulation of DCs with immunogenic or tolerogenic stimuli. Moreover, as a cellular energy sensor, AMPK is activated in response to low energy levels, such as an increased AMP:ATP ratio during nutrient deficiency or metabolic stress, in order to maintain intracellular ATP homeostasis. Activation of AMPK primarily results in the inhibition of protein synthesis by suppressing the activity of key translational regulators, such as p70S6K or 4E-PB1. Hence, it was also of interest to unravel to what extent energy requirements are implicated in DC specialization.

Since AMPK is activated by phosphorylation at T172 in its activation loop, we analyzed this phosphorylation and found that its intensity remained rather unchanged following stimulation of DCs with tolerogenic IL-10, TGFβ, and SSP effectors. However, stimulation with GM-CSF + IL-4 or LPS resulted in its reduction (Fig. 3D). Nevertheless, this reduction did not seem to impact mTOR signaling as the phosphorylation of TSC2 at S1387, a target site for AMPK, was not altered.

AMPK can inhibit mTOR signaling in two distinct ways (Fig. 3C). Firstly, activated AMPK phosphorylates TSC2 at S1387, thereby activating the activity of the GTPase-activating protein (GAP) of the TSC complex. Secondly, it directly phosphorylates Raptor in the mTORC1 protein complex at two well-conserved serine residues, which induces binding of the 14-3-3 protein to Raptor and leads to inhibition of the mTORC1 complex [10, 17, 32]. However, our data indicate that AMPK appears to have little to no influence on mTOR-mediated differentiation of DCs, neither in the immunogenic context studied here nor in tolerogenesis.

### GSK3β kinase is inhibited in DCs upon both immunogenic and tolerogenic stimulation

In subsequent experiments, we examined the influence of GSK3β (glycogen synthase kinase-3 beta) on the mTOR-mediated specialization of DCs in response to tolerogenic and immunogenic stimuli. GSK3β is a fascinating kinase with regard to the regulation of the mTOR cascade. It is a multifunctional protein kinase that interacts with the mTOR cascade in various ways. It can both activate and inhibit it. For example, GSK3β interacts with the PI3K/Akt signaling pathway, resulting in phosphorylation of GSK3β at S9 and its inhibition, which leads to the activation of mTORC1. Conversely, GSK3β can phosphorylate and inhibit Akt [33], leading to the suppression of mTORC1 activity. Additionally, it has been reported that activation of the canonical Wnt pathway, which is always associated with the inhibition of GSK3β, stimulates the mTOR signaling cascade [34]. Thus, the activation of the GSK3β kinase leads to the suppression of mTOR signaling, and GSK3β inhibition is essential for mTORC1 activation [35]. And indeed, numerous kinases have been reported to exert inhibitory phosphorylation on GSK3β at the S9 position. In addition to Akt, these include p70S6K, RSK, PKC, PKA, and Wnt signaling [36]. In fact, GSK3β is known to engage in the crosstalk with several signaling pathways, suggesting that it may serve as a coordination hub, integrating various cellular signals to modulate the mTOR pathway. For example, activated GSK3β can phosphorylate the downstream effector of mTOR, the p70S6K at S371, abolishing its kinase activity [37]. However, activated p70S6K kinase is able to inhibit GSK3β in a feedback mechanism, for example in response to high amino acid content, to support protein synthesis [38, 39]. Hence, inhibition of mTOR signaling via GSK3β thus requires AMPK activity, while activation of mTOR in turn requires inhibition of GSK3β.

With this knowledge in mind, we examined the inhibitory phosphorylation of GSK3β at S9. It was indeed strongly phosphorylated and manifested earlier and more persistently following stimulation with GM-CSF + IL-4 and LPS compared to stimulation with IL-10, TGFβ, or SSPs (Fig. 3D), mirroring the phosphorylation pattern of p70S6K. Consistent with this inhibitory phosphorylation, we did not observe any alterations in p70S6K phosphorylation at S371, a recognized target site for GSK3β.

Thus, these results further confirmed the activation of the mTOR signaling cascade following both immunogenic and tolerogenic stimulations, and the distinct pattern of its activation following the two different types of differentiation signals, but did not elucidate the potential upstream triggers of these stimulations. One potential inducer could be canonical Wnt signaling, an involvement of this pathway in mTOR activation has been documented previously [5, 10, 35, 40]. Indeed, active Wnt signaling is recognized as one of the most potent inhibitors of GSK3β, and it has been reported that Wnt signaling not only directly facilitates the phosphorylation of p70S6K, S6, and 4E-PB1, but also suppresses the phosphorylation of TSC2 by GSK3β in coordination with AMPK [34]. Therefore, we investigated the phosphorylation of S675 and S552 of β-catenin, the primary effector protein of the Wnt cascade, which no longer undergoes degradation after activation of Wnt signaling, but accumulates and translocates to the nucleus, where it regulates gene transcription. Phosphorylation of β-catenin at these sites, mediated by PKA and Akt, respectively, enhances its accumulation in the nucleus and its transcriptional activity [41]. However, we were unable to detect either changes in phosphorylation at these sites or accumulation of β-catenin in the cytosol or nuclei (Fig. 3D and Additional file 1, Fig. S2D) following either immunogenic or tolerogenic activation of the mTOR cascade, suggesting that Wnt signaling hardly plays any role in the events analyzed here.

### PI3K is the key activator for both immunogenic and tolerogenic specification of DCs

**Fig. 4:**
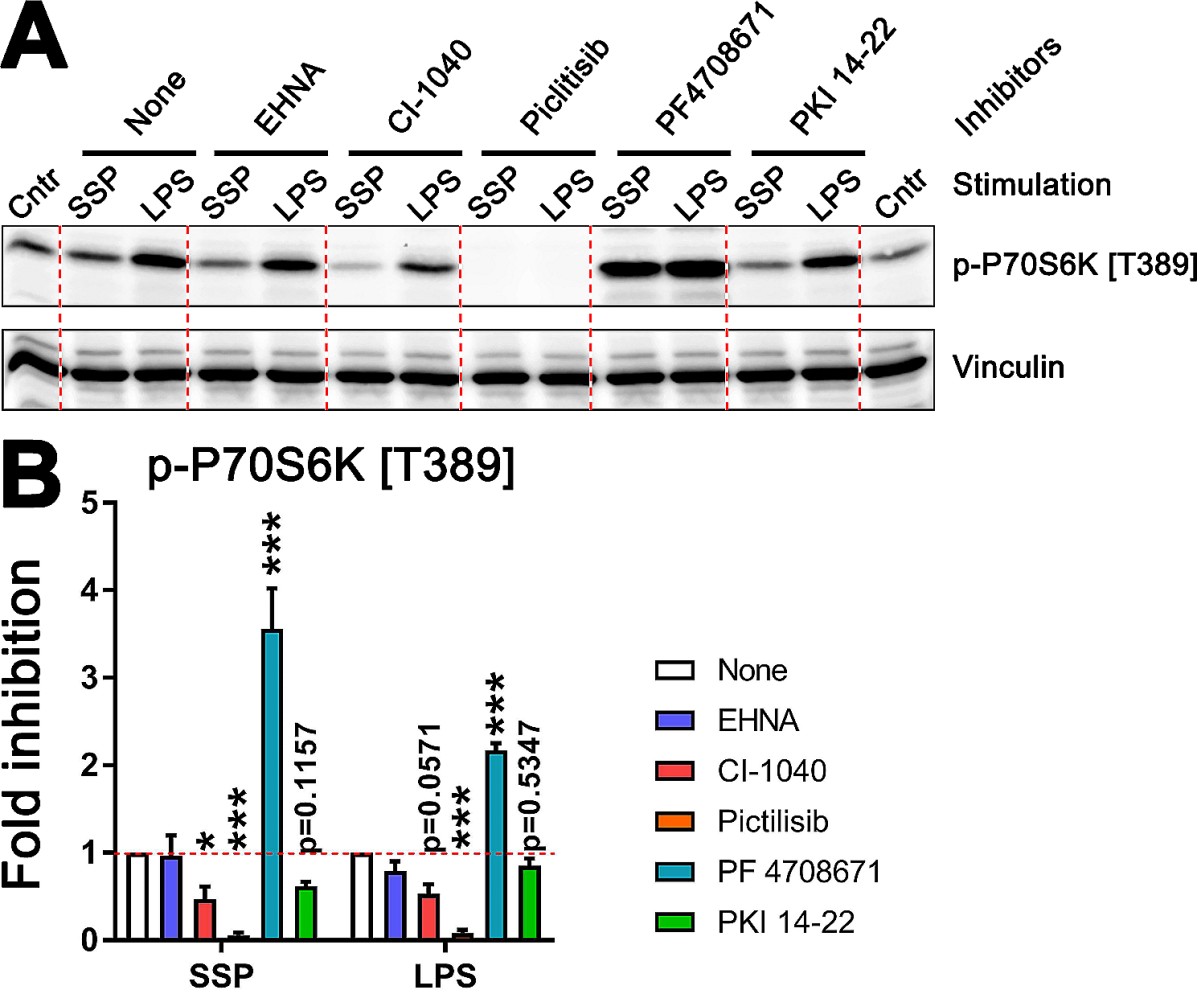
Analysis of p70S6K activation phosphorylation in DCs after stimulation with SSPs or LPS in the presence of different kinase inhibitors. (**A**) A representative Western blot showing phosphorylation of p70S6K at T389. EHNA is an inhibitor for adenosine deaminase, CI-1040 for ERK1/2, pictilisib for PI3K, PF4708671 for p70S6K and PKI-14-22 for PKA. (**B**) Quantification of p70S6K phosphorylation in response to SSPs (left panel) and LPS (right panel) from three independent experiments. Phosphorylation intensity of p70S6K in cells stimulated with either SSPs or LPS but without inhibitors were arbitrarily set to 1. Mean values ± SEM are shown. Statistical analysis was performed using two-way ANOVA followed by Tukey multiple comparison. *P < 0.05, **P < 0.01, and ***P < 0.001

And finally, as a proof of concept, we aimed to investigate the potential effect of inhibiting specific components of the mTOR cascade or its potential activators on p70S6K phosphorylation in response to immunogenic or tolerogenic stimuli, potentially resulting in different outcomes. To this end, we chose SSPs for the tolerogenic stimulus and LPS for the immunogenic stimulus. Semi-mature DCs deprived of GM-CSF + IL-4 cytokines were starved for 6 h in RPMI medium with 1% FCS before being exposed to SSPs or LPS in the presence of chemical inhibitors targeting adenosine deaminase (EHNA), ERK1/2 (CI-1040), PI3K (Pictilisib), p70S6K itself (PF4708671), or PKA (PKI-14-22) for a duration of 4 h. Subsequently, the cell lysates were examined for phosphorylation of p70S6K at T389 (Fig. 4A). The optimal inhibitor concentrations were established in preliminary experiments, and the DCs were consistently pre-treated with the inhibitors 30 min prior to the addition of SSPs or LPS to remove any kinase activity in the unstimulated cells.

Inhibition of adenosine deaminase by EHNA is known to result in an accumulation of free adenosine, a potent trigger of tolerogenesis [31], but it had little effect on DCs stimulated with SSPs or LPS (Fig. 4A and B). However, inhibition of ERK or PI3K kinases significantly reduced the SSP- and LPS-induced phosphorylation of p70S6K, underscoring their importance for mTOR activation in response to both tolerogenic and immunogenic agents. Interestingly, inhibition of p70S6K kinase with F4708671 led to the accumulation of activating phosphorylation at T389, seemingly because the inhibitory compound hinders downstream activation of phosphatases, thereby maintaining the persistent phosphorylation state, a phenomenon also observed with the inhibition of other kinases such as p38 kinase [42]. Inhibition of PKA had a marginal effect. PKA is a known inhibitor of GSK3β and could, in theory, be involved in the regulation of mTOR kinase. Thus, these inhibitory experiments highlighted the impact of PI3K and ERK signaling on mTOR activation following both immunogenic and tolerogenic stimulation of DCs.

In summary, of the mTOR upstream kinases analyzed here (Fig. 2D), three appear to be relevant for SSP-mediated stimulation of the mTOR cascade: Akt, ERK and GSK3β. The first two were found to be activated, while the latter was inactivated. On the other hand, IKKs and AMPK do not seem to be involved. Additionally, both the activation of Akt and ERK, as well as the inactivation of GSK3β, occur through the PI3K pathway, as evidenced by the effective inhibition of the first two kinases and the activation of GSK3β upon treatment with pictilisib, which does not affect AMPK-activating phosphorylation (Fig. S2B and C).

The exact mechanism by which PI3K is activated by the diverse stimuli examined in this study remains unknown and warrants further investigation. But it is rather unlikely that all stimuli activate the PI3K/Akt cascade via the same receptors and according to the same principle. While the activation of the PI3K/Akt signal transduction pathway by LPS and GM-CSF is well established and known to be directly mediated by toll-like receptor-4 (TLR4) and Janus kinase 2 (JAK2) signaling, respectively [43, 44], the activation of the pathway by tolerogenic stimuli is less well studied and rather indirect. TGFβ has been shown to activate the pathway in conjunction with the PDGFB receptor [45, 46] and available data suggest that in IL-10-initiated signaling, AMPK acts upstream of PI3K, specifically activating the p55 regulatory subunit of PI3K [47, 48]. However, in our experiments, we could not detect any changes in AMPK activating phosphorylation at T172 after IL-10 stimulation (Fig. 3D). Regarding SSPs, our recent publication revealed that their main constituents are different thymosins, with thymosin beta 4 being the most abundant component [49]. Thymosin beta 4 has indeed been shown to stimulate the PI3K signaling pathway by binding to the ILK-PINCH complex [50].

Another intriguing aspect of the activation of the mTOR cascade by SSPs could be the involvement of adenosine receptors. In our recent study, we demonstrated that SSPs, in contrast to immunogenic stimuli, promote tolerance by inducing a significant de novo synthesis of extracellular ATP in DCs [49], potentially through binding to the ecto-ATP synthase [51]. Extracellular ATP is rapidly converted to adenosine, and inhibition of adenosine receptors resulted in a reduced formation of immunosuppressive Treg cells by DCs in response to SSPs, while leaving the induction of immunogenic Tbet-positive T cells unchanged [49].

The four adenosine receptors A1, A2A, A2B, and A3 belong to the class of purinergic G-protein-coupled receptors. Subtypes A1 and A3 signal mainly via Gi proteins, leading to the inhibition of adenylyl cyclase and PKA, while subtypes A2A and A2B signal mainly via Gs proteins, leading to the activation of adenylyl cyclase and the stimulation of cyclic adenosine monophosphate (cAMP) formation as well as the activation of PKA [52–54]. However, the intracellular signaling pathways triggered by adenosine receptors are more complex and include, in addition to adenylyl cyclase, phospholipase C, inositol triphosphate, diacylglycerol, PI3K, and MAPKs [54–57, 54, 56, 57].

**Fig. 5:**
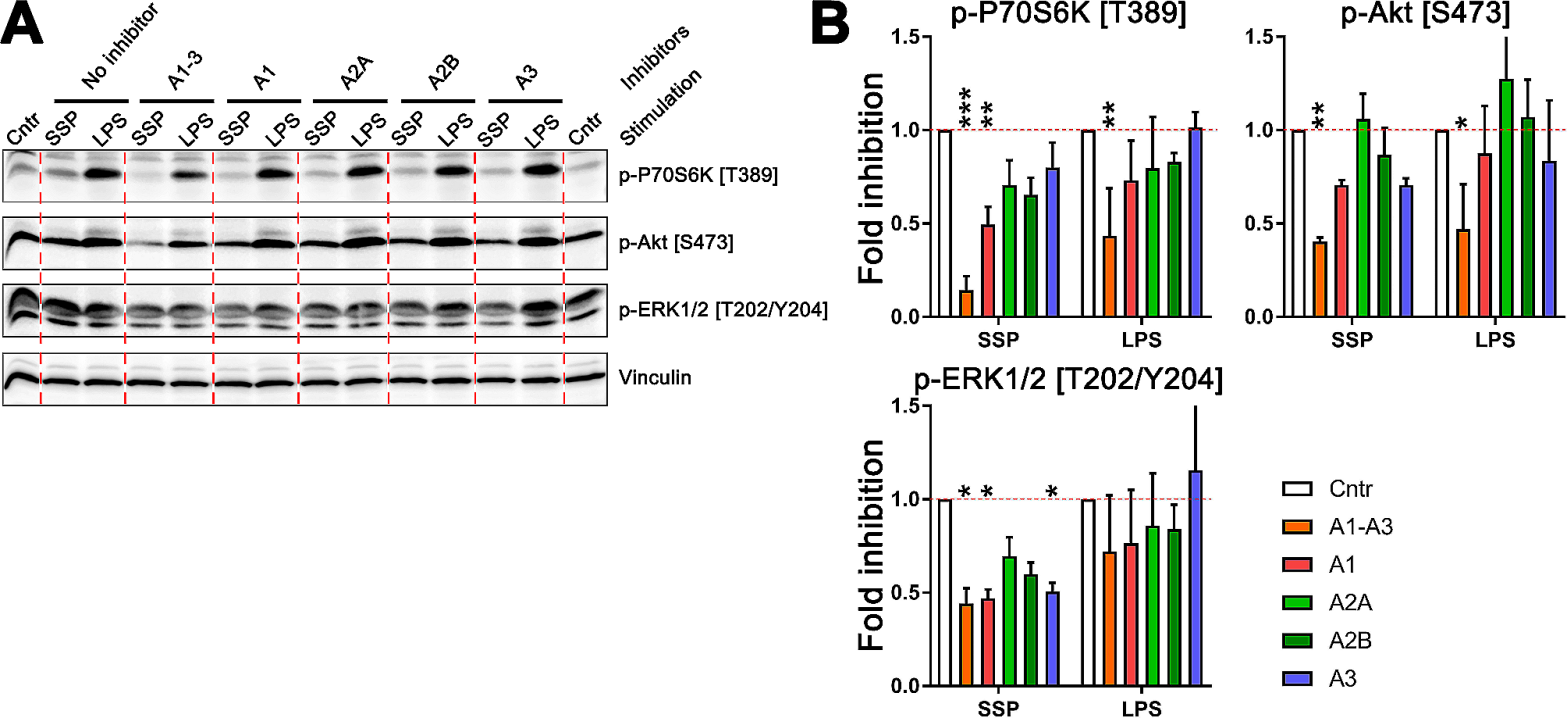
Analysis of mTOR activation phosphorylation in DCs after stimulation with SSPs or LPS in the presence of adenosine receptor inhibitors. (**A**) Representative Western blots showing phosphorylation of p70S6K at T389, Akt at S473 and ERK1/2 at T202/Y204. The following adenosine receptor inhibitors were used: CGS15943 (pan inhibitor) at 5 µM, DPCPX (A1R inhibitor) at 10 µM, ZM241385 (A2AR inhibitor), and MRS1706 (A2BR inhibitor) at 100 nM, and MRS1523 (A3R inhibitor) at 10 µM. (**B**) Quantification of p70S6K (left panel), Akt (right panel) and ERK1/2 (lower panel) phosphorylation in response to SSPs and LPS from three independent experiments. The phosphorylation intensities of the kinases in cells stimulated with either SSPs or LPS but without inhibitors were arbitrarily set to 1. The mean values ± SEM are shown. Statistical analysis was performed using two-way ANOVA followed by Tukey multiple comparison. The significance levels are indicated as follows: *P < 0.05, **P < 0.01, and ***P < 0.001

Therefore, we exposed starved semi-mature DCs to either SSPs or LPS in the presence of chemical inhibitors that target adenosine receptors for a duration of 4 h. Following this exposure, we assessed the phosphorylation levels of p70S6K at T389, Akt at S473, and ERK 1/2 at T202/Y204 in the cell lysates (Fig. 5). Overall, the inhibition of adenosine receptors had a stronger inhibitory effect on phosphorylation induced by SSP stimulation compared to LPS stimulation. Interestingly, only the pan inhibitor of adenosine receptors demonstrated a significant reduction in the phosphorylation of p70S6K and Akt kinases following LPS exposure of DCs. However, this reduction was less pronounced compared to the effect observed after SSP exposure, indicating differential impacts of the two stimuli on the phosphorylation of p70S6K and Akt kinases in DCs. Specifically, the phosphorylation levels of p70S6K at T389 remained at 14% for SSP stimulation, whereas for LPS stimulation, the phosphorylation levels remained at 43%. On the other hand, the application of specific inhibitors targeting individual adenosine receptors did not exhibit significant inhibitory activity on the phosphorylation of LPS-stimulated DCs. Conversely, in DCs exposed to SSPs, the inhibition of both A1 and A3 receptors resulted in a significant reduction in kinase phosphorylation. The lack of effect of A2 receptor inhibitors that communicate via pKA signaling is consistent with the results in Fig. 4, which show that inhibition of PKA does not significantly affect SSP-induced mTOR activation. These results suggest that SSPs may effectively initiate the activation of the mTOR cascade through adenosine receptors, while this may not be the case for LPS, indicating distinct responses based on the stimulus.

In summary, both immunogenic and tolerogenic stimuli activate the mTOR signaling cascade in DCs via the PI3K pathway. Nonetheless, the exact mechanisms underlying the initiation of PI3K activation by these stimuli are not fully understood and require further investigation. The specific steps involved in this process are likely to be diverse, reflecting the complexity of the mechanisms underlying PI3K activation by different stimuli. Furthermore, our experiments revealed that SSPs elicit mTOR cascade stimulation similar to classical tolerogenic stimuli such as IL-10 and TGFβ, underscoring their tolerogenic properties. However, SSPs not only mimic or contain the two cytokines, but also stimulate the mTOR cascade in a unique manner, indicating a distinctive mode of action. Considering that SSPs are natural splenic peptides with the ability to restore impaired peripheral tolerance and prevent autoimmune diseases [12], the data presented here seem to be of particular importance and emphasize the central role of the mTOR pathway in tolerance formation in particular and in the restoration of lost peripheral immunological tolerance in general. Another key finding of this study is that both immunogenic and tolerogenic stimuli activate the mTOR signaling cascade, suggesting that the disparity between immunogenesis and tolerogenesis is not qualitative but rather quantitative. Immunogenic stimuli prompt rapid, robust, and sustained activation of the mTOR cascade, whereas tolerogenic stimuli induce this response with a time delay and less intensity, albeit still activating the signaling pathway.

### Stimulation of DCs with SSPs supports high mitochondrial activity characteristic of tolerogenic DCs

**Fig. 6:**
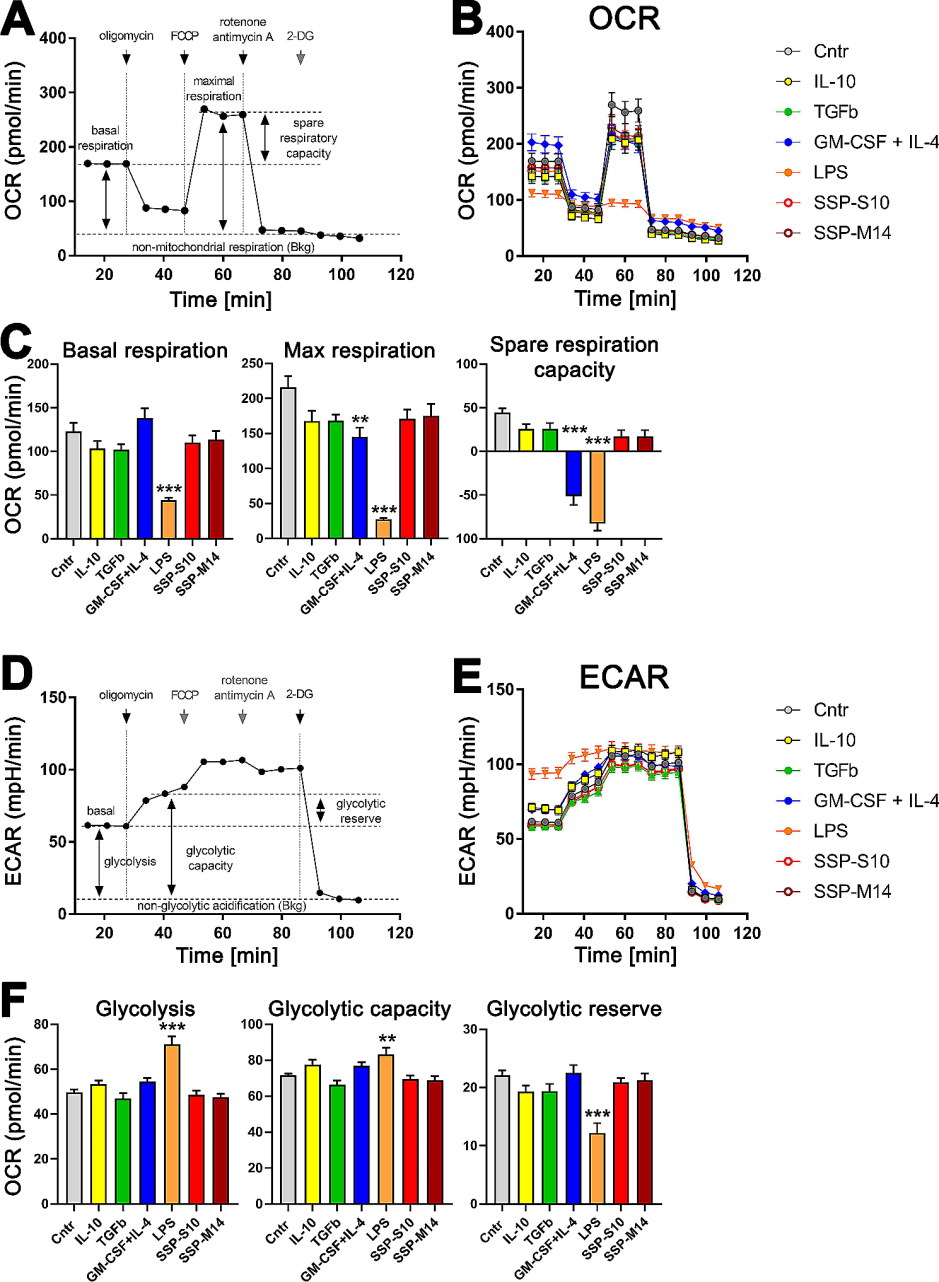
Comparison of the metabolic phenotype of DCs stimulated with classical immunogenic (GM-CSF + IL-4, LPS) or tolerogenic (IL-10, TGFβ) stimuli versus SSPs in real-time using the Seahorse Mito Stress Test assay. (**A**) Schematic representation of main mitochondrial respiration in real-time. Shown is a typical OCR curve for basal respiration and after addition of oligomycin (complex V inhibition), FCCP (maximal respiration induction) and rotenone/antimycin A mixture (electron transport chain inhibition). (**B**) Representative real-time curves of OCR of mitochondria in DCs stimulated with the indicated agents. (**C**) OCR quantification of basal respiration (left panel), maximal respiration (middle panel) and spare respiratory capacity (right panel) from four independent experiments. (**D**) Schematic representation of the main real-time glycolysis. Shown is a typical ECAR curve for the extracellular acidification rate in the basal state and after addition of oligomycin (induces cell glycolysis by inhibiting mitochondrial complex V) and after2-DG (2-deoxy-D-glucose, a glycolysis inhibitor). (**E**) Representative real-time curves of glycolysis-dependent ECAR in DCs stimulated with the indicated agents. (**F**) ECAR quantification of glycolysis (left panel), glycolytic capacity (middle panel) and glycolytic reserve (right panel) from four independent experiments. Shown are mean values ± SEM. Statistical analysis was performed using one-way ANOVA followed by Tukey’s multiple comparison. *P < 0.05, **P < 0.01, and ***P < 0.001

The mTOR signaling pathway not only regulates immune responses but also orchestrates energy metabolism, serving as a central regulator that balances anabolic and catabolic processes to maintain metabolic equilibrium in response to nutrient availability. Notably, alterations in energy metabolism are intimately associated with the differentiation of cells into immunogenic or tolerogenic state. While immunogenic stimuli tend to trigger a transition from mitochondrial oxidative phosphorylation (OXPHOS) to aerobic glycolysis, a process known as the Warburg effect, tolerogenic DCs or immature DCs predominantly rely on OXPHOS metabolism [58, 59]. Furthermore, tolerogenic and immature DCs exhibit enhanced adaptability in their bioenergetic capacity for ATP synthesis, enabling them to dynamically adjust their metabolism to meet the demands of their specialized functions [59–61]. Given the close association between the metabolic phenotype of DCs and their activation and functional specialization, we wondered whether stimulation of immature DCs with SSPs would also elicit metabolic changes characteristic of tolerogenic DCs. The plausibility of this interest is further supported by our analysis of the mTOR pathway and previous research demonstrating that the shift of immature DCs from oxidative phosphorylation to aerobic glycolysis by LPS is mediated by the PI3K/Akt signaling pathway and counteracted by AMPK [58]. We therefore analyzed the oxygen consumption rate (OCR, Fig. 6A, B and C) and extracellular acidification rate (ECAR, Fig. 6D, E and F) of SSP-stimulated DCs in comparison to classical immunogenic (GM-CSF + IL-4, LPS) and tolerogenic (IL-10, TGFβ) stimuli in real time using the Seahorse Mito Stress Test assay. For this purpose, freshly isolated bone marrow cells were incubated with GM-CSF + IL-4 for 6 days to obtain semi-immature DCs, and after washing off the cytokines, the cells were stimulated with SSPs, tolerogenic (IL-10, TGFβ) or immunogenic (LPS, GM-CSF + IL-4) cytokines for a further 48 h before being used in the assay.

As expected, we observed no differences in the maximal respiration in DCs stimulated with IL-10 and TGFβ or SSPs after addition of FCCP (disrupts mitochondrial membrane potential and cause a collapse in the proton gradient), regardless of whether the peptides were derived from porcine (SSP-S10) or murine (SSP-M14) spleen. In contrast, stimulation of the cells with immunogenic stimuli resulted in lower maximal respiratory activity (Fig. 6B). Quantification of the results of four replicate experiments confirmed that immunogenic stimulation of immature DCs resulted in lower OCR than stimulation with tolerogenic stimuli or SSPs (Fig. 6C). A particularly strong reduction in OXPHOS was observed in cells treated with LPS, a potent classical tolerogenic stimulant. Interestingly, the most striking difference between immunogenic and tolerogenic stimuli was found in spare respiratory capacity, which represents the difference between basal and maximal respiration and characterizes the mitochondrial capacity to meet additional energy demands under acute cellular stress to avoid an ATP crisis [61]. Real-time analysis of glycolysis also showed that LPS-stimulated DCs had the highest glycolytic activity and therefore the lowest glycolytic reserve capacity, indicating that only strong immunogenic stimuli lead to the induction of glycolysis, while tolerogenic stimuli, including SSPs, did not significantly alter the glycolytic function of DCs (Fig. 6E, F), which is in good agreement with previously published data [58, 59]. Of note, our data show that although stimulation of DCs with GM-CSF + IL-4 definitely drives them towards immunogenesis, their metabolic phenotype is not as advanced as in end-differentiated LPS-stimulated DCs, suggesting residual metabolic plasticity and potential reprogramming. It is also worth noting that cytokine deprivation in immature DCs shifts them towards tolerogenesis. Culturing these cells for two days without any stimulus did not lead to apoptosis, as our previous studies showed [12], but conferred tolerogenic DCs capabilities in terms of mitochondrial oxidative respiration (Fig. 6A-C, Cntr sample), but also in terms of their ability to generate Tregs from immature CD4^+^ cells in co-culture, where they were as efficient as IL-10- TGFβ- or SSP-stimulated DCs [12].

Collectively, these findings suggest that SSPs predominantly exert a tolerogenic effect, aligning with the results obtained from the analysis of the mTOR signaling pathway. Indeed, the data of this work reinforce our previous observations, demonstrating that SSPs confer tolerogenic properties to immature DCs and further emphasize the significant role of SSPs as natural regulators of peripheral immunological tolerance [12]. In addition, our data confirmed that tolerogenic DCs display greater metabolic plasticity, indicating their ability to efficiently adjust their energy production and biosynthetic needs to environmental or physiological changes. This adaptability is reflected in the distinct types of mTOR pathway stimulation observed.

Thus, in addition to the cellular mechanisms of SSP-mediated tolerogenesis that have been reported recently [12], we have taken the first step toward elucidating the molecular mechanisms of this fascinating and promising phenomenon. Our next endeavor is to decipher transcriptomic changes induced by SSP stimulation in order to decipher the metabolic phenotypes of DCs stimulated by SSPs compared to canonical tolerogenic and immunogenic stimuli, aiming to gain further molecular details of SSP-induced tolerogenesis.

### Electronic Supplementary Material

Below is the link to the electronic supplementary material.

Supplementary Material 1

## Data Availability

No datasets were generated or analysed during the current study.

## Abbreviations

2-DG: 2-deoxyglucose
4E-PB1: eukaryotic initiation factor 4E-binding protein 1
Akt: Akt serine/threonine kinase or protein kinase B alpha
AMPK: AMP-activated protein kinase
DCs: dendritic cells
Deptor: DEP domain containing mTOR interacting protein
ECAR: extracellular acidification rate
eIF-4E: eukaryotic initiation factor 4E
ERK: extracellular-signal Regulated Kinase
FCCP: carbonyl cyanide-4 (trifluoromethoxy) phenylhydrazone
FCS: fetal calf serum
GM-CSF: granulocyte-macrophage colony-stimulating factor
GSK3β: glycogen synthase kinase 3 beta
IKK: I-kappa-B-kinase
IL-10: interleukin 10
IL-4: interleukin 4
ILK: integrin linked kinase
JAK2: Janus kinase 2
LPS: lipopolysaccharide
mTOR: mammalian target of rapamycin
mTORC: mTOR complex
NF-κB: nuclear factor kappa-light-chain-enhancer of activated B cells
OCR: oxygen consuption rate
OXPHOS: oxydative phosphorylation
p70S6K or (S6K1): 70 kDa ribosomal protein S6 kinase 1 or (ribosomal protein S6 kinase B1)
PDGFB: Platelet-derived growth factor, beta subunit
PDK: 3-phosphoinositide dependent protein kinase
PI3K: phosphatidylinositol 3-kinase
PINCH or LIMS: LIM and senescent cell antigen-like-containing domain protein
PKA: pritein kinase A
pRAS40: 40 kDa proline-rich AKT substrate
Raptor: regulatory associated protein of MTOR
Reb: Ras homolog enriched in brain
Rictor: rapamycin-insensitive companion of mTOR
S6: S6 ribosomal protein
SSPs: small spleen peptides
TGFβ: transforming growth factor beta
TLR4: toll like receptor 4
Treg: regulatory T cells
TSC: Tuberous sclerosis complex
