# Supplementary material for: Conversion of dendritic cells into tolerogenic or inflammatory cells depends on the activation threshold and kinetics of the mTOR signaling pathway
Source: Cell Commun Signal. 2024 May 21;22:281. doi: 10.1186/s12964-024-01655-1 (PMC11106905; doi:10.1186/s12964-024-01655-1)
Supplement: Supplementary file 1 — Supplementary Material 1 [file 12964_2024_1655_MOESM1_ESM.docx]

**Additional file 1**

**
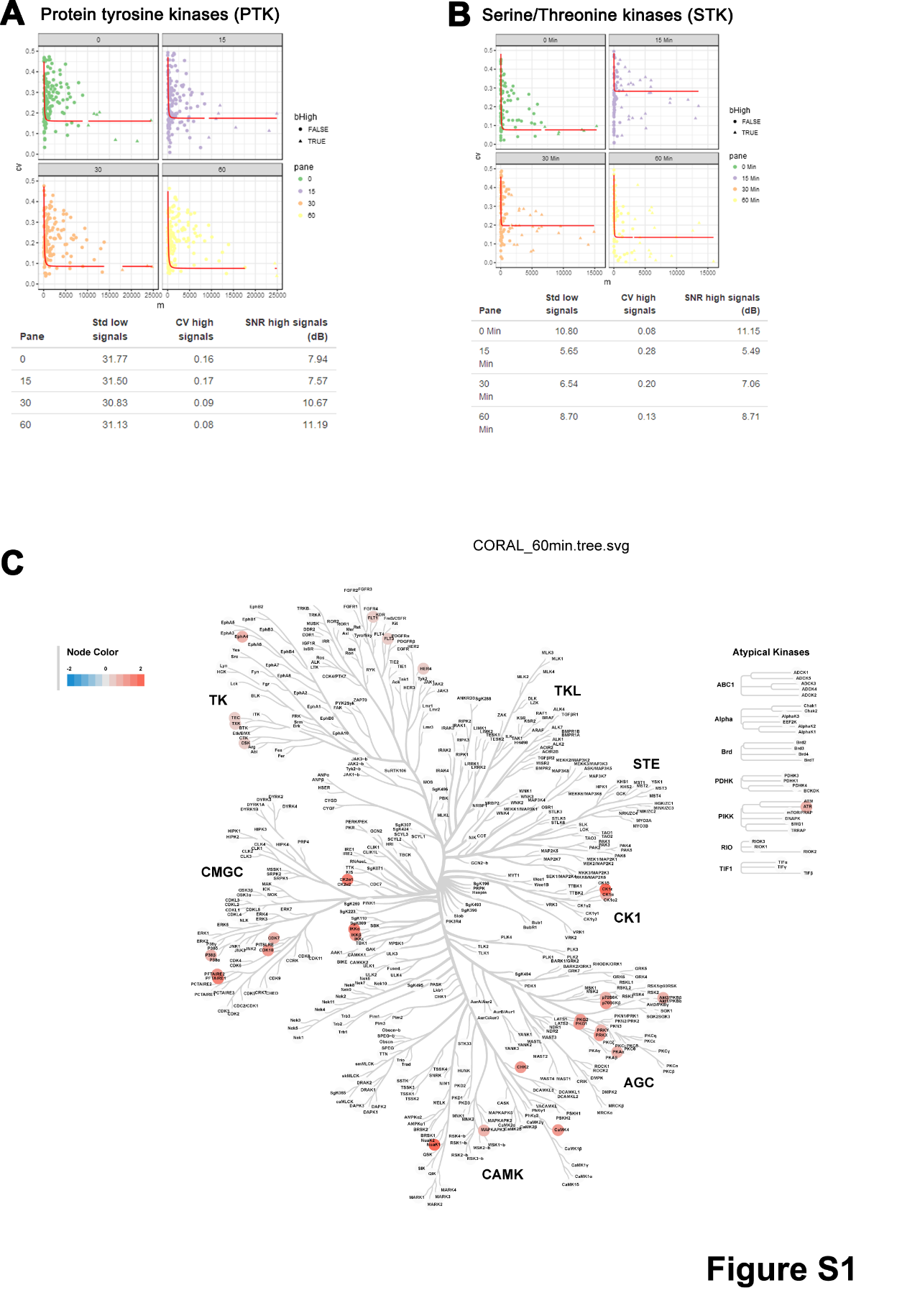
**

**Fig. S1.** Kinase selectivity profile of DCs stimulated with SSPs for 0, 15, 30 and 60 minutes. Biological samples used for kinase activity profiling show low variance. DCs were stimulated with SSPs for 15, 30 or 60 min and the activity of tyrosine kinases (**A**) and serine/threonine kinases (**B**) was assessed by chip‐based kinase activity profiling (PamGene technology). Depicted are coefficient of variance (CV) plots of three independent experiments. (**C**) Phylogenetic tree for protein kinases with color of nodes indicating their activity. Only the phylogenetic tree of active kinases in DCs revealed after 60 min of SSP stimulation is shown.


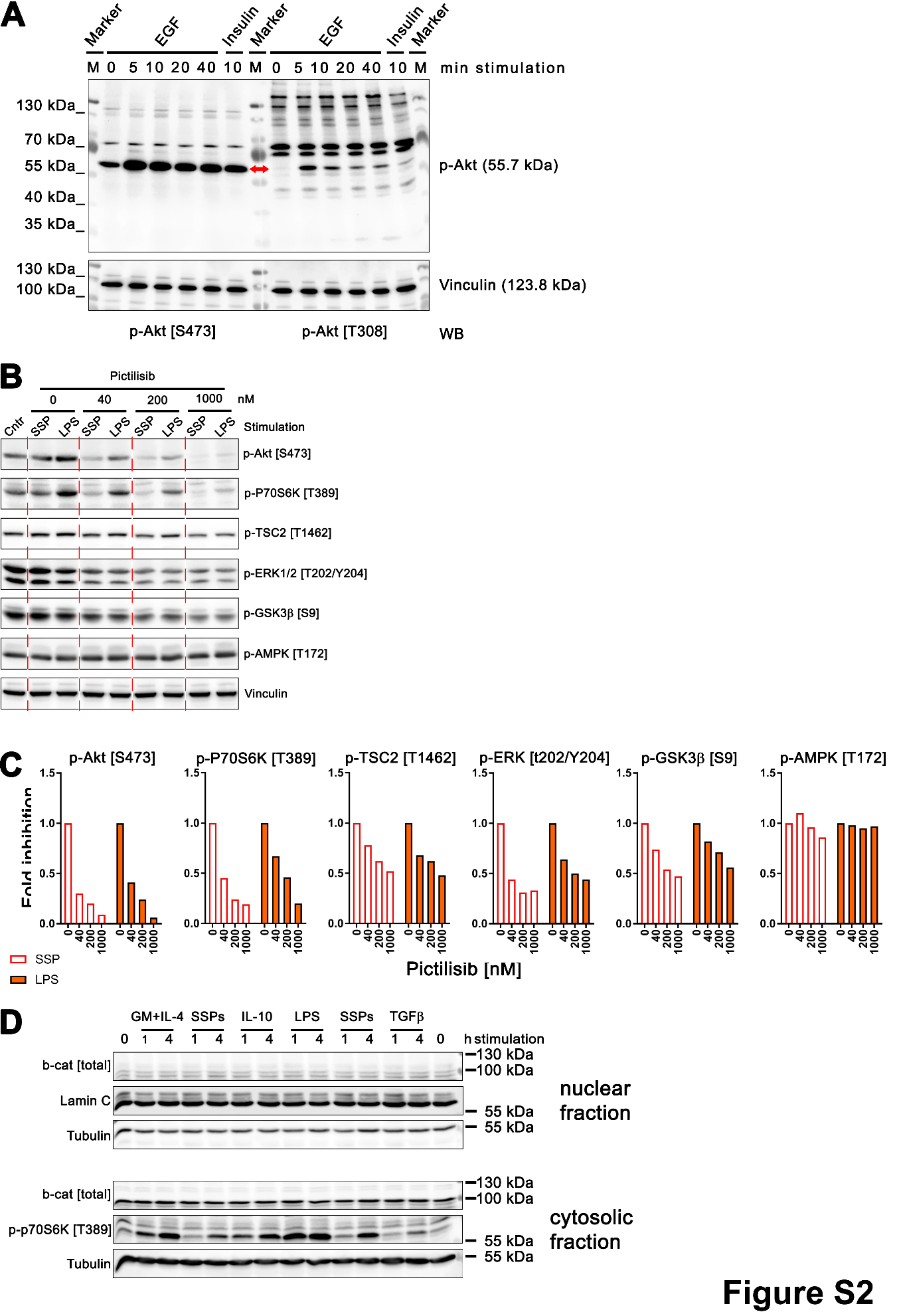


**Fig. S2.** (**A**) Testing the efficiency and specificity of antibodies that recognize the phospho-S473 and phospho-T308 sites of Akt kinase. Starved A549 cells were stimulated with 100 ng/ml EGF or insulin for the indicated times, and equal amounts of cell lysates were separated by SDS-PAGE. Samples were loaded onto a gel twice in the same order, and after electroblotting, the left half of the membrane was analyzed for the phospho-S473 site and the right half of the membrane was analyzed for the phospho-T308 site. The vinculin blot served as a loading control. Representative blots of two independent experiments are shown.

(**B**) The PI3K inhibitor pictilisib effectively inhibits the stimulation of the mTOR cascade by both SSPs and LPS. GM-CSF+IL-4-matured DCs were starved for 6 hours and stimulated with SSPs (5 µg/ml) or LPS (50 ng/ml) in the presence of different concentrations of the PI3K-specific inhibitor pictilisib for 4 hours. Cells were pre-incubated with the inhibitor 30 minutes prior to stimulation with SSPs or LPS to enhance the inhibitor efficiency. After separation of the cell lysates by SDS-PAGE, the samples were analyzed for phospho-sites on the indicated proteins. Vinculin blot served as loading control. Representative blots of two independent experiments are shown.

(**C**) Quantification of the phosphorylation intensity of the proteins shown in (B). The phosphorylation intensity of the corresponding protein in cells stimulated with either SSPs or LPS, but without inhibitors, was arbitrarily set to 1.

(**D**) β-catenin does not accumulate in the cell nucleus, neither after stimulation of DCs with immunogenic nor tolerogenic stimuli. Starved semi-mature DCs were stimulated for 1 or 4 hours with the agents indicated above the panels. After separation of the cell lysates into nuclear and cytosolic fractions, equal amounts of lysates were separated by SDS-PAGE and analyzed for the presence of β-catenin. Blots for lamin C and tubulin served as loading controls for nuclear and cytosolic fractions. Phosphorylation of p70S6K at T389 served as a functional control for the stimuli used.


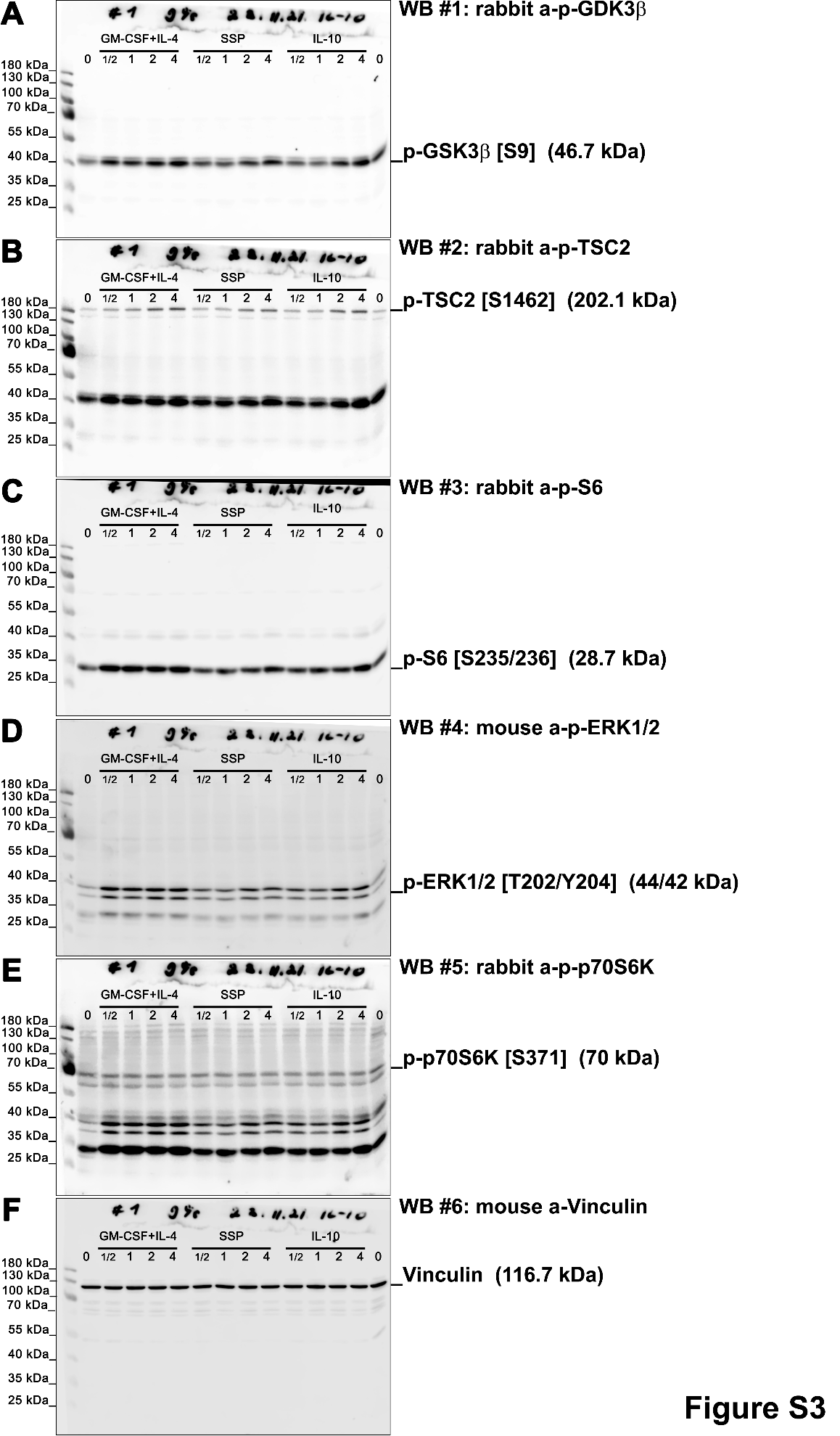


**Fig. S3.** Uncropped Western blot images. (**A-F**) The six blots presented here demonstrate the underlying principle behind the protein analysis conducted using the Western blot method. Each nitrocellulose membrane was employed to examine a range of 5 to 7 distinct proteins. Sequential Western blotting was carried out without stripping the antibodies from previous blots. However, careful consideration was given to ensuring that the proteins under analysis were not located close to each other in terms of their molecular weight and subsequent position on the membrane. It should be noted that the order in which the proteins were detected on the respective membranes did not always align with the sequence depicted in the figures of this manuscript.
